# Eating habits and carotenoid skin content among children based on their attendance at the school meals: A cross-sectional pilot study

**DOI:** 10.1016/j.jcte.2024.100378

**Published:** 2024-11-23

**Authors:** Giovanna Caparello, Fabrizio Ceraudo, Francesca Meringolo, Giuseppina Augimeri, Giuseppe Morino, Daniela Bonofiglio

**Affiliations:** a Department of Pharmacy, Health and Nutritional Sciences, University of Calabria, 87036 Arcavacata di Rende, CS, Italy; b Nutrition Unit, Bambino Gesù, Children’s Hospital IRCCS, Rome, Italy; c Centro Sanitario, University of Calabria, Via P. Bucci, Arcavacata Di Rende (CS), 87036, Rende, Cosenza, Italy

**Keywords:** Mediterranean Diet, Nutrition, Food education program, Carotenoid score, Veggie Meter, Fruits, Vegetables

## Abstract

**Objective:**

The promotion of a healthy diet, such as the Mediterranean Diet (MD), among childhood is of a particular importance, since eating behaviors learned early in life have been shown to be maintained into adolescence and adulthood. The most efficient intervention in childhood is the active involvement of the schools.

**Design:**

The aim of this study was to evaluate the adherence to the MD model and the skin carotenoid levels among children divided by their school lunch attendance.

**Methods:**

This cross-sectional study involved 132 pupils (64 girls and 68 boys), divided between children who ate lunch at school (44%) and at home (56%). The children who had meals provided by the school participated in activities promoting the health benefits of fruits and vegetables. All participants underwent anthropometric measurements and assessment of the MD adherence and the physical activity using KIDMED and PAQ-C questionnaires, respectively, and skin carotenoid content using the Veggie Meter®.

**Results:**

We found mean KIDMED and PAQ-C scores, while skin carotenoid content was below the normal range in our population sample. Interestingly, children who ate lunch provided by the school had significantly higher carotenoid scores with respect to those who had lunch at home (*p = 0.005*). In multiple regression analyses, we found that carotenoid scores were positively influenced by gender (*p = 0.03*), school lunch attendance (*p = 0.01*) and daily vegetable consumption (*p = 0.0002*) in our children population sample.

**Conclusions:**

Our results suggest the importance of promoting a healthy lifestyle at the school to improve eating habits during childhood as a strategy for disease prevention across the lifespan.

## Introduction

Childhood is an important period of life, during which lifestyle and a correct diet play a crucial role in the development of healthy adults. The increasing prevalence of childhood obesity is associated with the emergence of comorbidities, including type-2 diabetes mellitus, dyslipidemia, hypertension, nonalcoholic fatty liver disease and obstructive sleep apnea. The most common cause of obesity in children is a positive energy balance due to caloric intake exceeding calorie expenditure combined with a genetic predisposition to weight gain [1]. Eating habits and lifestyle factors represent the most important modifiable risk factors for obesity [2]. In recent years, it has been observed an increase in the portion sizes and the consumption of high-energy foods and a decrease in fruit and vegetable consumption along with a reduction in physical activity levels and an increase in the sedentary behavior in the pediatric population [3], [4]. Socio-economic factors have been found to influence the access to healthy foods and the opportunity to carry out physical activity, supporting the development of obesity [5].

Preventing childhood obesity requires a multisectoral approach involving families, schools, communities and public policies [6]. Family-based lifestyle interventions, including dietary modifications and increased physical activity, are the cornerstone of weight management in children [7].

Regarding the schools, some effective strategies include nutrition education campaigns that promote the importance of a balanced diet which limit the consumption of high-calorie, low-nutrient foods along with initiatives that encourage regular physical activity among children, including school sports programs, parks accessible games and urban policies favorable to physical and sporting activities.

Within a perspective of healthy dietary habits and lifestyle, the Mediterranean Diet (MD), one of the healthiest dietetic patterns, traditionally consumed in countries that surround the Mediterranean Sea, represents a dietary pattern able to prevent the development of many diseases, in childhood to guarantee health benefits for the adulthood [8], [9]. It is not a simple diet, but a lifestyle based on the consumption of healthy foods, regular physical activity, adequate rest and conviviality. MD recommends a high intake of plant foods, comprising mainly fruits and vegetables, whole grains, legumes, nuts, and seeds; locally grown, fresh, seasonal, unprocessed foods; olive oil as the main source of fat; low to moderate amounts of cheese and yogurt; low quantities of meat and meat products; high quantities of fish; and low to moderate amounts of red wine with meals for adults [8]. It has been widely demonstrated the importance of fruit and vegetable intake, foods placed at the basis of the MD pattern, due to their health benefits, especially in the young population. Indeed, fruits and vegetables are enriched in several micronutrients, including carotenoids, which prevent the development of several chronic diseases [10], [11]. Adequate fruit and vegetable consumption is crucial for healthy children to grow, yet consumption often remains low, especially in this young population [12], [13]. Interventions aimed to increase fruit and vegetables consumption in children should be widespread in the school and community settings [14], [15]. Despite the numerous benefits, there are often difficulties in implementing these eating habits in developmental age, such as the availability of fresh foods and resistance to dietary changes on the part of young people. However, involving children in food decisions, educating them about the benefits of the MD and offering a variety of tasty dishes could help change unhealthy eating habits that lead to excess weight [16]. Therefore, the aim of this study was to evaluate the adherence to the MD model and the skin carotenoid levels among children divided by their attendance to the meals provided by the school.

## Materials and methods

### Study design

This cross-sectional study involved 132 pupils (64 girls and 68 boys), aged from 8 to 10 years and attending the elementary school “L. Plastina Pizzuti” of Cosenza, Calabria Region, Italy, who were recruited into the “Dammi il 5 Project”, carried out by the Department of Pharmacy, Health and Nutritional Sciences and by Health Center of the University of Calabria and promoted by “Associazione Pancrazio” located in Cosenza, Italy. The participants were divided, in compliance with gender equality, into two groups based on their habits of attending the lunch at the school or at home. The data were collected in May 2023. Criteria of exclusion from the study were metabolic and chronic diseases, liver diseases, any kind of cognitive or physical/motor limitation, any kind of restrictive diet (i.e., hypocaloric, low carbohydrate, and low fat). After obtaining informed consent from their parents, the children participated with the help of the parents in scientific research activities, which involved the collection of data regarding their food choices and physical and sporting activities through the compilation of questionnaires. Three nutritionists collected anthropometric parameters (weight and height) and measured the concentration of skin carotenoids through the use of a non-invasive instrument, the Veggie Meter ®. The “Dammi il 5” project was included in scientific research approved by the Ethic Committee of the University of Calabria, Italy (#53519/2022).

### Anthropometric parameters

Participants’ weights were determined using the TANITA BC-545 N with a load capacity of 150 kg and an accuracy of 100 g. Height was determined using a Seca stadiometer, with a maximum capacity of 220 cm and an accuracy of 1 mm. Body mass index (BMI) and the Z-score was calculated as previously reported [17].

### Questionnaire of adherence to the Mediterranean diet (KIDMED)

Adherence to the MD was assessed administering the validated Mediterranean Diet Quality Index (KIDMED) questionnaire. The KIDMED comprises 16 items, with 4 questions reflecting unfavorable dietary habits (consumption of fast food, baked goods, sweets, and skipping breakfast), scored with a value of − 1 each, and 12 questions denoting a positive connotation (consumption of oil, fish, fruits, vegetables, cereals, nuts, pasta or rice, dairy products, and yogurt), scored with a value of + 1 each. The resulting KIDMED score ranges from < 0 to ≤ 12, with higher scores representing a higher adherence to the MD [18].

### Children’s physical activity questionnaire (PAQ-C)

The Physical Activity Questionnaire for Children (PAQ-C) provides general estimates of physical activity levels [19]. Briefly, the 7-day recall self-administered questionnaire included information on participation in different types of activities and sports collected in nine items. Each item ranges from 1 (very low) to 5 (very high) score by which the average score represents the PAQ-C score.

### Skin carotenoid level measurement by Veggie Meter®

Skin carotenoid levels were measured using the Veggie Meter® (Longevity Link Corporation, Salt Lake City, UT, USA, http://www.longevitylinkcorporation.com/products.html, accessed on 18 September 2023), a spectroscopy-based device according to the manufacturer’s instructions [20]. Briefly, the device’s calibration was performed with the provided dark and white reference materials prior to the data collection. Then, the skin carotenoid measurement was executed on the index finger of the non-dominant hand of each child. The subjects inserted their finger into the device applying a modest pressure in order to decrease the blood perfusion of the measured tissue volume, which might interfere with the measurement of skin carotenoid content. A computer analyzed the light that was reflected from the finger and provided a score on a spectral range from 0 to 800, with a higher score indicating higher skin carotenoid stores. Information relating to the subject such as age, sex, weight, and height was entered into the appropriate software.

### Recreational initiatives and promotion of the Mediterranean diet model

**Fig. 1:**
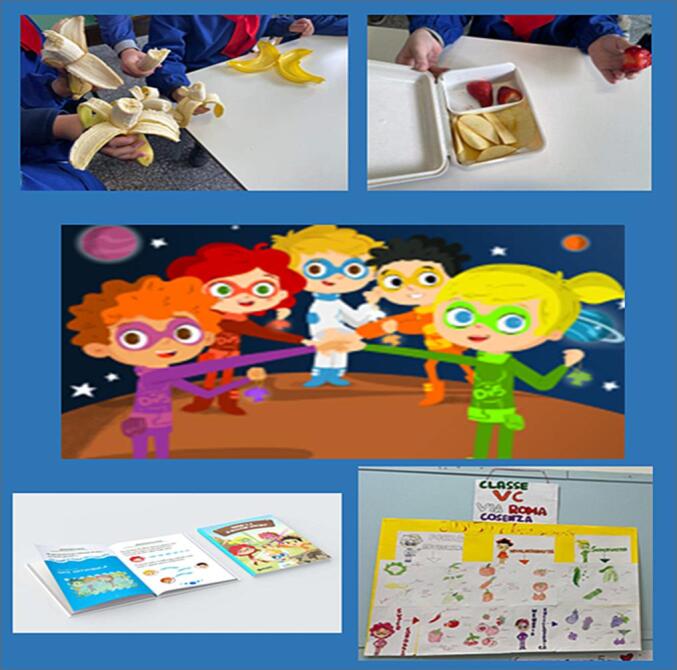
Recreational activities in the context of the “Dammi il 5 Project”.

The children having meals at the school participated in individual and group recreational activities (before, during and after lunchtime) with the nutritionists of the “Dammi il 5” project carried out in several schools of Southern Italy, as a best practice example of a nutritional education program from January 2023 to June 2024. Specifically, this program, based on MD recommendations, consisted in presentations with multimedia slides, drawings, preparation of posters and simple quizzes relating to healthy eating, where the theme of the five colors of health, mainly represented by seasonal varieties of fruits and vegetables, were pictured as five superheroes, protagonists of a series of cartoons and books (Fig. 1).

### Statistical analysis

Data were reported as the mean and standard deviation (SD). Statistical differences were evaluated using parametric tests (Student’s *t*-test and Chi-square test) in GraphPad-Prism 7.00 software program, as applicable. Multivariate linear regression model was analyzed using GraphPad-Prism 7.00. Sample size calculation was performed using a 95 % confidence interval (CI) and a margin of error (d) of 5 %. A minimum number of 120 participants from primary schools was requested. Statistical significance was set at *p* < 0.05.

## Results

### General characteristics of the schoolchildren population

A total of 132 elementary schoolchildren, 64 girls and 68 boys aged between 8 and 10 years old participated in our cross-sectional study. In Table 1, we reported the anthropometric characteristics along with KIDMED, PAQ-C and carotenoid scores of the total study population or categorized on the habits of attending lunch at the school (44 % of total population sample) or at home (56 % of total population sample). The mean values of each variable studied in the children population categorized by sex and according to their habits of having lunch at the school or at home are reported in Supplementary Table 1. As shown in Table 1, the mean values of anthropometric parameters were within the normal range in the total population sample, with no differences between children attending lunch at the school or at home. Moreover, we found a medium degree of adherence to the MD pattern evaluated by questionnaires on eating habits (mean KIDMED: 5.33 ± 2.36) and physical activity (mean PAQ-C: 2.72 ± 0.65) in the total population sample with no changes in the two groups (Table 1). It is worth to note that our children population had the mean of skin carotenoid content, evaluated using the Veggie Meter®, below the normal range (300.66 ± 97.42) (Table 1), without gender differences (Supplementary Table 1). Interestingly, children having meals at the school had significantly higher carotenoid scores respect to those had lunch at home (327.09 ± 102.8 *vs* 279.66 ± 88.1, *p = 0.005*) (Table 1), which remain evident in both sexes (girls: 310.03 ± 81.23 *vs* 275.06 ± 92.76, *p = 0.05*; boys: 348.08 ± 122.8 *vs* 283.24 ± 85.28, *p = 0.002*) (Supplementary Table 1). We also observed that the attendance to the school lunch was influenced by the health status of the children’s parents and the educational level of the children’s mothers (Table 1). Moreover, differences were found in the habit of having dinner with the TV at home (45 % *vs* 66 %, *p = 0.003*) and in the parents’ perception of their children’s lean body weight (10 % *vs* 20 %, *p = 0.05*) and normal (38 % *vs* 63 %, *p = 0.0004*) as well as active (50 % *vs* 31 %, *p = 0.006*) physical activity according to the attendance of the school lunch (Table 1).

**Table 1:** General and parent–child demographic characteristics of total children population sample and based on their attendance at the school lunch.

		**Attendance at the school lunch**		
**Characteristics**	**Total**	**Yes**	**No**	***p*-Value**
Subjects (n) (%)	132	58 (44)	74 (56)	0.09
Age (yrs)	8.16 ± 0.81	8.1 ± 1.11	8.27 ± 0.44	0.23
Weight (Kg)	32.10 ± 2.36	32.40 ± 7.83	31.87 ± 7.33	0.69
Height (cm)	132.55 ± 8.99	131.51 ± 8.58	133.36 ± 9.27	0.24
BMI (kg/m^2^)	18.3 ± 3.49	18.54 ± 3.19	17.88 ± 3.71	0.35
BMI Z-score	−0.42 ± 0.83	0.81 ± 2.82	0.51 ± 1.45	0.20
KIDMED score	5.33 ± 2.36	5.05 ± 2.33	5.55 ± 2.38	0.23
PAC-Q score	2.72 ± 0.65	2.65 ± 0.75	2.77 ± 0.57	0.33
Carotenoid score	300.66 ± 97.42	327.09 ± 102.80	279.66 ± 88.1	0.005
**Parent-child demographic characteristics**				
*Health status (%)*				0.001
Healthy	51	64	40	
Diabetes, obesity, hypercholesterolemia, ictus or stroke	45	35	53	
Not reported	4	1	7	
*Instruction grade (%)*				
Elementary				¥0.26
Father	1	2	0	°0.54
Mother	0	0	0	§0.002
Middle school				
Father	13	16	10	
Mother	12	15	9	
High school				
Father	52	50	52	
Mother	40	53	30	
University				
Father	24	24	24	
Mother	39	27	49	
Not reported				
Father	10	7	12	
Mother	9	5	12	
	**Eating and lifestyle habits**			
Are vegetables prepared every day at home and for dinner? (%)	51	50	51	0.89
Is fruit always on the table at home and for dinner? (%)	60	67	69	0.76
Is fruit offered as a snack as an alternative to snacks (sweet or savory) at home? (%)	84	84	84	1
Are legumes prepared at least 3 times a week at home? (%)	60	64	57	0.31
Is fish prepared at least 3 times a week at home? (%)	37	40	35	0.46
Does the child show interest in preparing lunch and dinner dishes? (%)	80	79	81	0.72
Do you have dinner with the TV at home? (%)	57	45	66	0.003
Is there a TV in the child's room? (%)	58	59	58	0.88
	**Parental perception of child body weight and physical activity**			
How do you consider your child weight? (%)				
Lean	16	10	20	0.05
Normal weight	71	76	68	0.21
Over weight	8	7	9	0.60
Fat	2	3	0	0.08
How do you consider your child for the physical activity? (%)				
Normal	52	38	63	0.0004
Active	40	50	31	0.006
Lazy	5	7	3	0.19

¥ total father *vs* mother in total population °father yes *vs* father no §mother yes *vs* mother no.

Then, multiple regression analysis was performed using the carotenoid score as dependent variable and sex, KIDMED, BMI Z score, PAQ-C along with the school lunch attendance as independent variables. Interestingly, the results showed that the carotenoid score was correlated with gender (*p = 0.03*) and positively associated with the habit of having school lunch in our children population (*p = 0.04*) (Table 2).

**Table 2:** Multiple regression analysis among carotenoid score and different variables in our children population sample.

**Variable**	***β* (95 % CI)**	**SE**	***p*-Values**
Sex [F]	−40.56 (−77.82; −3.31)	18.78	0.03
KIDMED	3.39 (4.312;11.09)	3.88	0.38
BMI Z SCORE	7.63 (0.594; 15.86)	4.15	0.07
PAQ-C	1.54 (−27.23; 30.32)	14.50	0.91
School lunch [YES]	40.29 (2.69; 77.89)	18.95	0.04
Adj.R2	0.08		

Adj: Adjusted, β: regression coefficient, CI: Confidence Interval, F: females, SE: standard error.

### Impact of the Mediterranean diet food choices on the carotenoid score in our children population sample

**Fig. 2:**
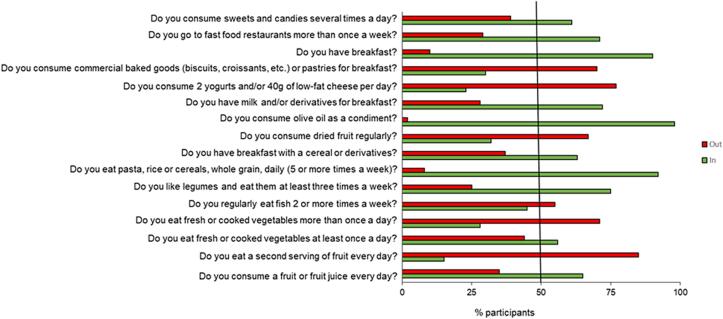
Compliance with items from the KIDMED questionnaire in the total sample children population. Percentage distribution of population with respect to the cut-of points within or outside recommendations according to KIDMED score.

Using the KIDMED questionnaire, we estimated the compliance rates for each MD recommendation in the total children population (Fig. 2) and categorized by sex (Supplementary Table 2).

As shown in Fig. 2, regarding the items on fruit and vegetable intakes only 15 % and 29 % children declared to eat a second serving of fruit every day and to eat fresh or cooked vegetables more than once a day, respectively. We also observed that 65 % and 56 % of children declared to consume a fruit or fruit juice every day and to eat fresh or cooked vegetables at least once a day, respectively.

Finally, we performed multiple linear regression analysis using the carotenoid score as the dependent variable and the habit to have lunch at the school along with the KIDMED items related to fruit and vegetable consumption as independent variables, in our children population sample. Intriguingly, we observed that carotenoid score was positively associated with the habit of eating lunch at the school (*p = 0.01*) and with the consumption of vegetables once a day (*p = 0.0002*) in our population (Table 3).

**Table 3:** Multiple regression analysis among Carotenoid score and different variables in our children population sample.

**Variable**	***β* (95 % CI)**	**SE**	***p*-values**
School lunch [YES]	40.86 (8.71; 73.1)	16.25	0.01
A fruit a day [YES]	−12.46 (47.62; 22.71)	17.77	0.48
Vegetables once a day [YES]	71.80 (35.09;108.5)	18.55	0.0002
A second fruit every day [YES]	−17.05(−63.97;29.86)	23.70	0.47
A second portion of vegetables a day [YES]	−25.52 (66.28; 15.24)	20.59	0.22
Adj.R2	0.1369		

Adj Adjusted, β regression coefficient, CI Confidence Interval, SE standard error.

## Discussion

In this study, we found higher skin carotenoid content in children having lunch at the school and following the best practice example of a nutrition education program, entitled “Dammi il 5” project. Our data highlight the importance of promoting fruit and vegetable intakes as healthier eating habits in childhood.

The promotion of a sustainable healthy diet, such as the MD model, among children is of particular importance, since eating behaviors learned early in life have been shown to be maintained into adolescence and adulthood [21], [22]. In particular, the primary and most efficient intervention in childhood is the active involvement of the schools, which convey important messages to families through their pupils, raising awareness among children towards healthy and proper diet and increasing fruit and vegetable consumption [23]. Mounting evidence indicates that adequate intakes of fruits and vegetables, which are essential source of minerals, vitamins, phytochemicals and fibers, exert a protective role against a wide range of metabolic and chronic diseases, such as overweight/obesity, type 2 diabetes, hypertension and cardiovascular diseases, cancer [24], [25], [26], [27], [28], [29]. Indeed, the recommended consumption of fruits and vegetables should be 200 g and 300 g, respectively, distributed in about five servings per day or at least 400 g/day of both fruits and vegetables, as suggested by WHO [30]. Results from the WHO European Childhood Obesity Surveillance Initiative (COSI) conducted in 13 countries [30], showed that the percentage of children who did not eat fresh fruits or vegetables daily varied widely, ranging from 19 % to 82 % for fruits and from 26 % to 91 % for vegetables; and it was higher among children whose parents had a lower level of education [31]. As part of COSI, in Italy OKkio alla SALUTE is the National Surveillance System promoted and financed by the Italian Ministry of Health, coordinated by the Italian National Institute of Health, in collaboration with the Italian Regions and the Ministry of Education that collects information about weight status, eating habits and physical activity levels, and promotes healthy eating in primary schools. In 2023, OKkio alla SALUTE involved 2.578 primary schools of which 76 % of the sampled schools have a canteen, 48 % provide for the distribution of healthy foods, 84 % provide curricular nutritional education, 27 % involve parents in initiatives for healthy eating habits [32]. Regarding no daily consumption of fruits and/or vegetables, recent data indicated that collectively 25.9 % of Italian children eat fruits and/or vegetables less than once a day, while 35.8 % children living in Calabria Region had this bad eating habit [32]. “Dammi il 5” project was a voluntary nutrition education program carried out in the schools of Southern Italy, including Calabria Region, to promote the MD model based on fruit and vegetable consumption. Nutritionists highlighted the benefits of eating more fruits and vegetables, by sharing the animated adventures of five children's characters, who become superheroes after eating their favorite fruits and vegetables, from which they draw resources that can help them overcome any obstacle. The five superheroes have captured the attention and interest of children during lunchtime leading to the awareness of a healthy and proper diet and improving their nutrition. Our data showed that only 15 % and 29 % of our population sample Italian children living in Calabria Region declared to eat a second serving of fruit everyday children and to eat fresh or cooked vegetables more than once a day, respectively, while 65 % and 56 % of children declared to consume a fruit or fruit juice every day and to eat fresh or cooked vegetables at least once a day, respectively. In this study, we extrapolated fruit and/or vegetable intakes by the items of the KIDMED questionnaire which could represent a bias associated with the self-reported questionnaire. However, we also used the Veggie Meter® as a validated and non-invasive method for the detection of skin carotenoid levels, accurately reflecting an objective measurement of fruit and vegetable intakes in children and in adults [11], [33], [34]. Here, we observed that the mean of skin carotenoid content, evaluated using the Veggie Meter®, was below the normal range, with no gender-related differences. Controversial data were reported on the relationship between skin carotenoids and sex in children, due to different factors such as weight status, ethnicity, seasonal variation, age [35], [36], [37], [38], [39]. Interestingly, we found significantly higher levels of skin carotenoids in children having meals at the school than those who had lunch at home. Furthermore, in multiple regression analysis the carotenoid score resulted positively associated with the attendance of meals at the school, where the nutritional education campaigns on the benefits of fruit and vegetable intakes should be conducted. We also observed that skin carotenoids were inversely correlated in girls in multiple regression analysis, underlining the relationship between carotenoids and biological sex which deserves to be explored in future studies.

Regarding the adherence to the MD pattern, we found medium scores of KIDMED and PAQ-C questionnaires in our children population, regardless sex. A medium degree to the food recommendations and physical activity of the MD pattern was reported in adolescents [40] and adults [33], [41] from the same geographical area, indicating the need to promote eating and lifestyle habits among populations living in Mediterranean area toward an optimal compliance to the MD able to prevent the development of metabolic and chronic diseases.

In the context of the healthy educational program, the importance of the nutritional messages at the school emerged from our multiple regression analysis, since carotenoid score was positively influenced by consuming vegetables once a day and again by attending lunch at the school.

Further studies evaluating the impact of educational program at baseline and after the nutritional intervention should be carried out to provide deeper insight in this research area.

## Conclusions

In summary, our pilot cross sectional study highlights the importance to improve eating choices towards healthier diet and provide valuable input for initiatives of MD promotion along with national nutrition policies among schoolchildren population.

## Ethics approval and consent to participate

Parents of children were provided written informed consent prior to the children enrolment in the trial. This study was conducted according to the guidelines laid down in the Declaration of Helsinki and approved by the Ethic Committee of the University of Calabria, Italy (#53519/2022).

## Consent for publication

All authors read and approved the final manuscript and provide consent for publication.

## CRediT authorship contribution statement

**Giovanna Caparello:** Writing – original draft, Data curation, Conceptualization. **Fabrizio Ceraudo:** Writing – original draft, Data curation, Conceptualization. **Francesca Meringolo:** Writing – original draft, Data curation, Conceptualization. **Giuseppina Augimeri:** Methodology, Formal analysis. **Giuseppe Morino:** Validation, Supervision, Conceptualization. **Daniela Bonofiglio:** Writing – review & editing, Validation, Supervision, Data curation, Conceptualization.

## Funding

This work was supported by the Associazione Pancrazio (“Iniziative in cofinanziamento 2019 con i bambini” FONDAZIONE CON IL SUD #2019-COF-01626), and by the Department of Excellence (Italian Law. 232/2016) the Department of Pharmacy, and the Health and Nutritional Sciences, University of Calabria, Italy. The funder was not involved in the study design, collection, analysis, interpretation of data, the writing of this article or the decision to submit it for publication.

## Declaration of competing interest

The authors declare that they have no known competing financial interests or personal relationships that could have appeared to influence the work reported in this paper.
