# Supplementary material for: Eating habits and carotenoid skin content among children based on their attendance at the school meals: A cross-sectional pilot study
Source: J Clin Transl Endocrinol. 2024 Nov 23;38:100378. doi: 10.1016/j.jcte.2024.100378 (PMC11629320; doi:10.1016/j.jcte.2024.100378)
Supplement: Supplementary Data 1 [file mmc1.docx]

**Supplementary Table 1. General characteristics of the children population** **categorized by sex and according to the habit of attending lunch at the school.**

|  |  | **Attendance lunch at the school** | | | |
| --- | --- | --- | --- | --- | --- |
| **Characteristics** | **Total** | **Yes** |  | **No** | ***p*-Value** |
| Subjects (%)  Girl  Boys | 48  52 | 55  45 |  | 43  57 | 0.09 |
| Age (yrs)  Girl  Boys  ***p*-Value** | 8.09 ±0.85  8.22±0.78  0.36 | 7.93±1.10  8.11±1.15  0.60 |  | 8.25±0.44  8.28±0.45 | 0.13  0.49 |
| Weight (Kg)  Girl  Boys  ***p*-Value** | 31.55±7.64  32.61±7.44  0.42 | 32.15±7.34  32.70±8.52  0.79 |  | 30.96±8.00  32.57±6.79  0.32 | 0.44  0.93 |
| Height (cm)  Girl  Boys  ***p*-Value** | 132.37±8.76  132.71±9.25  0.83 | 131.16±7.80  131.92±9.60  0.74 |  | 133.59±9.61  133.19±9.11  0.85 | 0.17  0.50 |
| BMI Z-scores  Girl  Boys  ***p*-Value** | -0.74±1.14  -0.21±0.5  0.91 | 0.81±1.21  0.83±1.26  0.94 |  | 0.07±1.80  0.84±1.02  **0.02** | **0.02**  0.96 |
| KIDMED score  Girl  Boys  ***p*-Value** | 5.5±2.47  5.18±2.26  0.43 | 5.22 ±2.27  4.84±2.44  0.55 |  | 5.78±2.66  5.38±2.15  0.48 | 0.26  0.24 |
| PAC-Q  Girl  Boys  ***p*-Value** | 2.8±0.69  2.64±0.63  0.25 | 2.81±0.80  2.47±0.67  0.11 |  | 2.77±0.57  2.77±0.57  0.99 | 0.782  **0.02** |
| Carotenoid score  Girl  Boys  ***p*-Value** | 292.54±88.27  308.4±105.51  0.35 | 310.03±81.23  348.08±122.80  0.16 |  | 275.06±92.76  283.24±85.28  0.70 | **0.05**  **0.002** |

**Supplementary Table 2.** **Compliance with items from the KIDMED test in our children population categorized by sex.**

| Items | Yes  (%) | No  (%) |
| --- | --- | --- |
| Do you consume a fruit or fruit juice every day? | G:67 | G:33 |
|  | B:63 | B:37 |
| Do you eat a second serving of fruit every day? | G:17 | G:83 |
|  | B:13 | B:87 |
| Do you eat fresh or cooked vegetables at least once a day? | G:56 | G:44 |
|  | B:56 | B:44 |
| Do you eat fresh or cooked vegetables more than once a day? | G:33 | G:67 |
|  | B: 25 | B: 75 |
| Do you regularly eat fish 2 or more times a week? | G:44 | G:56 |
|  | B: 46 | B: 54 |
| Do you like legumes and eat them at least three times a week? | G:75 | G:25 |
|  | B: 75 | B: 25 |
| Do you eat pasta, rice or cereals (barley, spelt, oats, etc.), preferably whole grain, daily? | G:95 | G:5 |
|  | B: 88 | B: 12 |
| Do you have breakfast with cereal or derivatives? | G:61 | G:39 |
|  | B: 65 | B: 35 |
| Do you consume dried fruit regularly? | G:30 | G:70 |
|  | B: 35 | B: 65 |
| Do you consume olive oil as a condiment? | G:98 | G:2 |
|  | B:99 | B:1 |
| Do you have milk and/or derivatives for breakfast? | G:72 | G:28 |
|  | B:72 | B:28 |
| Do you consume 2 yogurts and/or 40g of low-fat cheese per day? | G:27 | G:73 |
|  | B:19 | B:81 |
| Do you consume commercial baked goods (biscuits, croissants, etc.) or pastries for breakfast? | G:64 | G:36 |
|  | B: 75 | B:25 |
| Do you have breakfast? | G:86 | G:14 |
|  | B:94 | B:6 |
| Do you go to fast food restaurants more than once a week? | G:27 | G:73 |
|  | B:31 | B:69 |
| Do you consume sweets and candies several times a day? | G:38 | G:63 |
|  | B:40 | B:60 |

G: girls; B: boys.
